# Arcsine‐based transformations for meta‐analysis of proportions: Pros, cons, and alternatives

**DOI:** 10.1002/hsr2.178

**Published:** 2020-07-27

**Authors:** Lifeng Lin, Chang Xu

**Affiliations:** ^1^ Department of Statistics Florida State University Tallahassee Florida; ^2^ Department of Population Medicine College of Medicine, Qatar University Doha Qatar

**Keywords:** arcsine‐based transformation, Bayesian model, generalized linear mixed model, meta‐analysis, proportion

## Abstract

Meta‐analyses have been increasingly used to synthesize proportions (eg, disease prevalence) from multiple studies in recent years. Arcsine‐based transformations, especially the Freeman–Tukey double‐arcsine transformation, are popular tools for stabilizing the variance of each study's proportion in two‐step meta‐analysis methods. Although they offer some benefits over the conventional logit transformation, they also suffer from several important limitations (eg, lack of interpretability) and may lead to misleading conclusions. Generalized linear mixed models and Bayesian models are intuitive one‐step alternative approaches, and can be readily implemented via many software programs. This article explains various pros and cons of the arcsine‐based transformations, and discusses the alternatives that may be generally superior to the currently popular practice.

## 1 BACKGROUND

Many research findings in the health sciences are presented in the form of proportions, such as disease prevalence, case fatality rate, a diagnostic test's sensitivity and specificity, among others.^1^, ^2^ Meta‐analyses have been increasingly used to synthesize proportions that are reported from multiple studies on the same research topic.^3^, ^4^, ^5^, ^6^, ^7^, ^8^, ^9^, ^10^ Many meta‐analyses of proportions are performed using conventional two‐step methods. First, a specific transformation is usually applied to each study's proportion estimate for better approximation to the normal distribution, as required by the assumptions of conventional meta‐analysis models.^11^ Second, the meta‐analysis is performed on the transformed scale, and the synthesized result is then back‐transformed to the original proportion scale that ranges from 0% to 100%. Of note, one may also directly synthesize proportions without any transformation; however, this approach is not optimal, because the proportion estimates may not be approximately normally distributed, especially for rare events and small sample sizes. The Wald‐type confidence intervals (CIs) of proportions may be even outside the range of 0% to 100%.^12^

**Figure 1:**
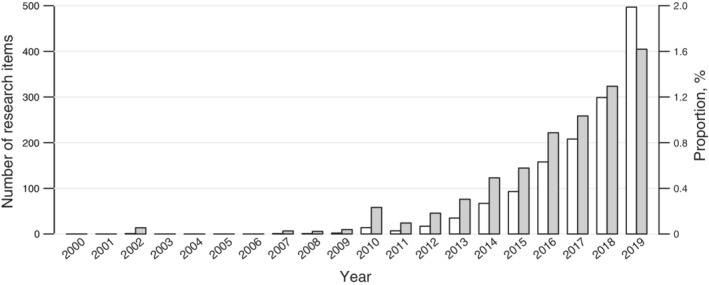
Bar plot of the number of research items using the double‐arcsine transformation in meta‐analyses and the corresponding proportion among meta‐analysis publications over the past two decades based on Google Scholar (https://scholar.google.com/). For each year, the left bar, in white, represents the number of research items, and the right bar, in gray, represents the corresponding proportion (in percentage)

Various transformations are available for proportions, including the log, logit, arcsine‐square‐root, and Freeman–Tukey double‐arcsine transformations.^13^, ^14^, ^15^, ^16^, ^17^ Among them, the Freeman–Tukey double‐arcsine transformation is a popular tool in current practice of synthesizing proportions.^10^ We did a search on Google Scholar on June 17, 2020; for each year between 2000 and 2019, we searched for the exact terms “meta‐analysis” and “double‐arcsine” to obtain the number of research items using the double‐arcsine transformation in meta‐analyses. We also searched for the exact term “meta‐analysis,” with restriction to article titles, to obtain the rough number of meta‐analysis publications in each year, and calculated the corresponding proportion of research items using the double‐arcsine transformation. Figure 1 shows that the double‐arcsine transformation has been increasingly used over the past two decades.

Despite the raising popularity, several authors have previously expressed concerns about arcsine‐based transformations.^18^, ^19^ In addition, many meta‐analyses do not even specify the transformation used for synthesizing proportions.^10^ Even if a transformation is specified, meta‐analysts frequently fail to provide sufficient justification for the selection of the transformation.

This article discusses the purported benefits of the arcsine‐based transformations that potentially explain their popularity in current practice. We also introduce how such transformations may be limited, and recommend alternative methods for meta‐analysis of proportions that may be superior. We focus on meta‐analysis of single proportions, where the arcsine‐based transformations are widely used.

## 2 METHODS

Suppose a meta‐analysis contains *N* studies that report single proportions on a common topic. Let *p*_*i*_ be the proportion estimate from study *i* in the meta‐analysis (*i* = 1, …, *N*). The proportion is then simply calculated as *p*_*i*_ = *e*_*i*_/*n*_*i*_, where *e*_*i*_ and *n*_*i*_ denote study *i*'s event count and sample size, respectively. The arcsine‐square‐root transformation is $y_{i}=g\left(p_{i}\right)=\arcsin{\sqrt{p}}_{i}$, with variance *v*_*i*_ = 1/(4*n*_*i*_). The Freeman–Tukey double‐arcsine transformation is

(1) $$y_{i}=g\left(p_{i}\right)=\text{arsin}\sqrt{e_{i}/\left(n_{i}+1\right)}+\text{arsin}\sqrt{\left(e_{i}+1\right)/\left(n_{i}+1\right)}$$

with variance *v*_*i*_ = 1/(*n*_*i*_ + 0.5).^13^ Of note, the formula above of the double‐arcsine transformation is the version originally presented in the article by Freeman and Tukey.^13^ While one may also take the average of the two arcsine values (by dividing by 2), leading to the variance *v*_*i*_ = 1/(4*n*_*i*_ + 2), so that it has the same scale with the arcsine‐square‐root transformation, such a linear transformation does not affect the back‐transformed proportion.

Besides the arcsine‐based transformations, the log and logit transformations are also frequently used for proportions.^10^, ^20^, ^21^ Their formulas are more straightforward: the log transformation is *y*_*i*_ = *g*(*p*_*i*_) = log*p*_*i*_, with variance *v*_*i*_ = 1/*e*_*i*_ − 1/*n*_*i*_, and the logit transformation is *y*_*i*_ = *g*(*p*_*i*_) = log[*p*_*i*_/(1 − *p*_*i*_)], with variance *v*_*i*_ = 1/*e*_*i*_ + 1/(*n*_*i*_ − *e*_*i*_).

After applying a specific transformation to each study's proportion, conventional meta‐analysis methods^22^ are subsequently performed using the transformed data, that is, *y*_*i*_ and *v*_*i*_, leading to the synthesized result *y* with a 95% CI. The synthesized result is finally back‐transformed to the original proportion scale; the overall proportion is usually estimated as *p* = *g*^−1^(*y*), and its CI limits are also back‐transformed in the same manner.

## 3 PROS

Because conventional meta‐analysis models assume normally distributed data,^11^ the various transformations are applied to the proportion data in an effort to yield better approximations to the normal distribution. As shown in the formulas above, the variances of the arcsine‐based transformations depend only on the sample sizes, which are typically fixed, known values. However, the variances of the log and logit transformations depend additionally on the event counts, which are random variables.

Involving event counts in the variances implies several limitations of the log and logit transformations. First, it does not meet the assumption of conventional meta‐analysis models; that is, the within‐study variances are treated as fixed, known values, while the event counts are not. The violation of this assumption may reduce statistical inference accuracy.^11^ Second, because the log‐ or logit‐transformed proportion estimates and their variances both depend on the event counts, they are intrinsically correlated. This intrinsic correlation has been well known to cause substantial biases in meta‐analytic results, especially when the sample sizes are small.^23^, ^24^, ^25^, ^26^, ^27^ In addition, in the presence of zero event counts, both log‐ and logit‐transformed proportions cannot be calculated, and a continuity correction must be applied to the zero counts, usually by adding 0.5.^28^, ^29^, ^30^ This correction may have considerable impact on the synthesized proportion for rare events.^31^

In this sense, the arcsine‐based transformations have the important advantage of stabilizing variances, which is likely the main reason that such transformations are widely used in current practice. As their variances depend only on the sample sizes, they can be validly treated as fixed, known values, and have no correlation with the transformed proportion estimates. These transformations also do not need the continuity correction for zero counts. Moreover, compared with the arcsine‐square‐root transformation, the Freeman–Tukey double‐arcsine transformation may stabilize variances better in general.^13^

## 4 CONS

Despite the advantages of the arcsine‐based transformations listed above, they also suffer from several critical limitations. First, these transformations lack intuitive interpretations from practical perspectives, especially compared with the traditional logit transformation.^18^, ^32^ The arcsine function is mainly used for technical purposes. More specifically, the variances of transformed proportions are approximated by the so‐called delta method, which uses the derivative of the transformation function.^33^ Taking the benefit of the special structure of the arcsine function's derivative, the event counts are canceled out in the approximated variances of the arcsine‐transformed proportions. Unlike the logit proportion that represents the odds on a logarithmic scale, the arcsine‐transformed proportions may not be intuitive for practitioners.^18^

Second, as the proportion estimates are usually heterogeneous, the random‐effects meta‐analysis method is frequently used, assuming that each study's underlying true transformed proportion follows the normal distribution across studies.^22^ The arcsine‐based transformations might violate this assumption, because the arcsine function has a bounded domain, implying truncations for the assumed normal distribution. On the other hand, the logit‐transformed proportions can take any real value, and thus, may be more suitable for the between‐study normality assumption.

Third, the Freeman–Tukey double‐arcsine transformation has a complicated form of back‐transformation to the original proportion scale. Compared with other transformations, its back‐transformation depends additionally on a sample size that represents the overall synthesized result.^34^ This “overall sample size” is not well defined in the meta‐analysis setting; it may be selected as the harmonic, geometric, and arithmetic means of study‐specific sample sizes,^19^, ^34^ or the inverse of the variance of the synthesized result^15^; it is generally difficult to justify the value used as the “overall sample size.” More importantly, different values may lead to substantially different proportions in some cases, potentially leading to misleading conclusions.^19^

Moreover, numerical problems may occur when using the Freeman–Tukey double‐arcsine transformation. Although this transformation refines the usual arcsine‐square‐root transformation by averaging over the double arcsines for better stabilizing variances, it may have low accuracy at values close to its domain limits, which likely occur in cases of rare events and small sample sizes.^35^ Specifically, because the event count *e*_*i*_ is between 0 and *n*_*i*_, as indicated in Equation (1), the transformed proportion must be bounded between $\text{arsin}\sqrt{1/\left(n_{i}+1\right)}$ and $\text{arsin}\sqrt{n_{i}/\left(n_{i}+1\right)}+\pi /2$. It is possible that the synthesized result is outside this domain based on a certain “overall sample size.” In this case, the result cannot be back‐transformed to the original proportion scale. When such issues occur, one may decide to use the back‐transformation of the arcsine‐square‐root transformation, which is a good approximation of the Freeman–Tukey double‐arcsine transformation for sufficiently large sample sizes; however, this might affect the accuracy of the analysis.

## 5 ALTERNATIVES

From a statistical perspective, event counts are typically assumed to follow binomial distributions,^36^ and all transformations discussed above are applied to the binomial data for approximations to normal distributions within studies. With advances in statistical computing techniques, these approximations in the two‐step methods may be unnecessary, because they can be feasibly replaced with one‐step meta‐analysis methods, including generalized linear mixed models (GLMMs) or Bayesian hierarchical models.^11^, ^18^, ^19^, ^37^, ^38^, ^39^, ^40^, ^41^, ^42^, ^43^

GLMMs directly model event counts with binomial likelihoods and fully account for within‐study uncertainties.^37^, ^38^, ^39^ They use a specific link function to transform study‐specific latent true proportion to a linear scale, on which random effects are specified in a manner similar to the conventional two‐step methods. The logit link is the canonical link function for proportions (ie, binomial data), while many other links, such as the log and probit links, may be also used.^36^ GLMMs with the log and logit links correspond to the log and logit transformations used in the two‐step methods, but they do not have any of the aforementioned limitations. Specifically, GLMMs do not involve estimating (transformed) proportions and their variances at the within‐study level. Therefore, they do not suffer from the problems caused by the intrinsic correlation between the log‐ or logit‐transformed proportions and their sample variances approximated by the delta method. GLMMs can also effectively model zero event counts without continuity corrections.^44^ More importantly, compared with the arcsine‐based transformations, the GLMM with the logit link produces more interpretable results.^18^, ^45^, ^46^, ^47^, ^48^

Similarly, the multilevel structure of meta‐analyses can be naturally modeled under the Bayesian framework. Bayesian methods assign priors to the unknown parameters, including the overall proportion and the heterogeneity variance on the transformed scale; the conclusions are drawn from the posterior distributions of these parameters. As one of the Bayesian methods' benefits, researchers might use informative priors to improve estimation by incorporating experts' opinion or external evidence.^49^

GLMMs and Bayesian models have been seldom used in meta‐analysis applications so far,^10^ despite the fact that the current literature offers many software programs to implement these alternative approaches for synthesizing proportions (as well as other measures), including SAS, R, and Stata.^39^, ^40^, ^45^, ^50^, ^51^, ^52^ Bayesian models can be fitted via BUGS, JAGS, Stan, and others that are designed for general purposes of Bayesian analyses.

When the number of studies or the number of events in a meta‐analysis is small (say, <10), GLMMs and Bayesian models may have issues about their algorithm convergence (more specifically, for maximizing likelihood and deriving posterior samples from the Markov chain Monte Carlo, respectively).^53^ In such situations, although the conventional two‐step methods might successfully produce results, the synthesized proportion may be subject to large biases and thus should be interpreted with great caution.^39^

As stated above, this article has focused on meta‐analysis of *single* proportions, where the arcsine‐based transformations are widely used. GLMMs and Bayesian methods are also available for jointly modeling *multiple* proportions, such as sensitivity and specificity of diagnostic tests.^45^, ^46^, ^47^, ^48^, ^54^

## 6 DISCUSSION

Compared with the traditional logit transformation, the arcsine‐based transformations for proportions mainly benefit from their stabilized variances that depend only on sample sizes. However, they do not have intuitive interpretations, and the limitations of the logit transformation can be easily overcome by using GLMMs or Bayesian models. These alternatives are straightforward, one‐step methods and are generally superior to the conventional two‐step methods that require transformations of proportions within studies.^55^ Importantly, in some cases, the one‐step methods may lead to substantially different results from the two‐step methods.^19^ In future studies, it is worthwhile to explore the performance of the different methods with various transformations or link functions based on a large collection of empirical meta‐analysis datasets, and quantitatively investigate the differences between the synthesized proportions produced by these methods.

The limitations of the arcsine‐based transformations *in meta‐analysis*, however, do not nullify their use *in individual studies*. We have focused on the synthesis of proportions, and GLMMs or Bayesian models are advantageous for producing such synthesized proportions. When meta‐analysts want to present estimates from individual studies, transformations of proportions are still useful. For example, meta‐analysts frequently use the forest plot to visualize the distributions of study‐specific estimates,^56^ and the funnel plot to assess potential publication bias or small‐study effects.^57^, ^58^ Both plots depend on each study's point estimate of proportion, CI, and SE, which cannot be obtained by GLMMs or Bayesian models. The arcsine‐based transformations can be preferably used at the within‐study level, while they are not recommended at the between‐study level. In fact, many early articles on the arcsine‐based transformations were discussed in the setting of an individual study^13^, ^59^, ^60^; these articles did not directly suggest extending the arcsine‐based transformations to the meta‐analysis setting.

In summary, we highly recommend the use of GLMMs or Bayesian models for synthesizing proportions; nowadays, many software programs are readily available for implementing them. Most meta‐analyses of proportions published in recent years continue to use the Freeman–Tukey double‐arcsine transformation, and the rate is increasing (Figure 1); it is a time for change.

## FUNDING

This research was supported in part by the U.S. National Institutes of Health/National Library of Medicine grant R01 LM012982 and National Institutes of Health/National Center for Advancing Translational Sciences grant UL1 TR001427. The content is solely the responsibility of the authors and does not necessarily represent the official views of the National Institutes of Health. The financial support had no involvement in the conceptualization of the report and the decision to submit the report for publication.

## CONFLICT OF INTEREST

The authors declare no conflict of interest.

## AUTHOR CONTRIBUTIONS

Conceptualization: Lifeng Lin, Chang Xu

Funding Acquisition: Lifeng Lin

Writing‐Original Draft Preparation: Lifeng Lin

Writing‐Review & Editing: Lifeng Lin, Chang Xu

All authors have read and approved the final version of the manuscript. Lifeng Lin had full access to all of the data in this study and takes complete responsibility for the integrity of the data and the accuracy of the data analysis.

## TRANSPARENCY STATEMENT

Lifeng Lin affirms that this manuscript is an honest, accurate, and transparent account of the study being reported, and that no important aspects of the study have been omitted.

## Data Availability

Data sharing is not applicable to this article as no new data were created in this study.
